# Rare Orbital Involvement Originating from Extranodal Marginal Zone Lymphoma

**DOI:** 10.3390/medicina60050706

**Published:** 2024-04-25

**Authors:** Yao-Chang Wen, Tzu-Chuan Huang, Wen-Chiuan Tsai, Shiue-Wei Lai

**Affiliations:** 1 Department of Internal Medicine, Tri-Service General Hospital, National Defense Medical Center, Taipei 114202, Taiwan; a40212a@gmail.com; 2 Division of Hematology/Oncology, Department of Internal Medicine, Tri-Service General Hospital, National Defense Medical Center, Taipei 114202, Taiwan; 3 Department of Pathology, Tri-Service General Hospital, National Defense Medical Center, Taipei 114202, Taiwan

**Keywords:** orbital tumor, orbital lymphoma, extranodal marginal zone lymphoma

## Abstract

Ocular adnexa region (OAR) primary lymphomas are uncommon, accounting for 1–2% of non-Hodgkin lymphomas and 8% of extranodal lymphomas. Extranodal marginal zone lymphoma (EMZL) originates from several epithelial tissues, including the stomach, salivary gland, lung, small intestine, thyroid gland, and ocular adnexa region. Here, we report a 66-year-old female patient who was diagnosed with EMZL of OAR. In consideration of the possible side effect of radiotherapy, such as conjunctivitis, visual acuity impairment, and even retinal complications, she received six cycles of triweekly targeted chemotherapy with rituximab, cyclophosphamide, vincristine, and prednisone (R-CVP) without radiotherapy. Then, she remained in complete remission up to the present day.

## 1. Introduction

Lymphomatous presence in the area around the eye (ocular adnexal region, OAR) is typically uncommon; however, it constitutes the most common ocular malignancy. Approximately 8% of extranodal lymphomas are estimated to originate in the OAR, where they are referred to as primary ocular tumors [1]. The secondary involvement of OAR with non-Hodgkin lymphoma (NHL) is estimated at 5%. Extranodal marginal zone lymphoma (EMZL) is the most common type of NHL to involve OAR, followed by follicular and diffuse large B-cell lymphoma [2].

EMZL is a slow-growing type of NHL that frequently presents with localized involvement and may involve multiple organs and tissues [3]. Symptoms related to the ocular region include blurred vision and floaters. Environmental factors, occupational exposure, autoimmune disorders, and infectious agents are some of the risk factors that can contribute to the development of ocular EZML. The age of onset for this condition typically manifests around the age of 65, predominantly affecting females. On average, there is a span of approximately 6 to 7 months between the emergence of symptoms and receiving a formal diagnosis. The clinical presentation of the condition exhibits significant diversity, largely contingent upon the specific anatomical site affected: around a quarter of cases present with conjunctival lesions, while the majority, constituting 75%, manifest as intraorbital masses. Moreover, bilateral involvement, affecting both eyes, is noted in approximately 10 to 15% of diagnosed cases [4]. Diagnosis can be established through ophthalmic examinations, contrast-enhanced computed tomography (CT), contrast-enhanced magnetic resonance imaging (MRI), positron emission tomography (PET), and tissue biopsy supported by the appropriate immunohistochemical staining [5].

Treatment for EMZL of OAR has not reached a consensus, with approaches based on retrospective case series and studies, such as radiotherapy, surgery, chemotherapy, and targeted therapy [5,6]. In this case report, we highlight the clinical journey of a 66-year-old woman presenting with unilateral eye symptoms. Initially misdiagnosed with conjunctivitis, her condition posed diagnostic challenges. However, through diligent investigation and clinical acumen, she was ultimately diagnosed with EMZL. This case underscores the importance of considering uncommon etiologies in refractory or atypical ocular presentations, emphasizing the necessity of thorough evaluation to ensure accurate diagnosis and timely intervention. She underwent successful treatment with targeted therapy and intravenous chemotherapy finally. A total of 18 months following the completion of treatment, there has been no recurrence of the disease observed up to the present day.

## 2. Case Report

A 66-year-old female patient presented with refractory infected conjunctiva of the right eye without keratoconjunctivitis sicca, local pain, itchy sensation, or abnormal discharge since February 2021, which had lasted for 1 year. In the past, she enjoyed robust health and was free from any systemic diseases. Furthermore, prior to this incident, she did not partake in the consumption of alcohol, betel nuts, or cigarettes. Her occupation as a housewife did not entail any exposure to jungle environments, and she had no history of contact with birds or animals. Her surrounding people do not have the same symptoms as her.

She was treated with antibiotics of ofloxacin 0.3% drops and artificial tears for bacterial conjunctivitis at a local clinic, which demonstrated a poor treatment response. Then, she came to our medical center for further examinations. The serum analysis is described as follows: white blood cell count 4.28 × 10^3^/µL (normal: 4.50–11.00/µL), hemoglobin 14.2 g/dL (normal: 12.0–16.0 g/dL), platelet count 226 × 10^3^/µL (normal: 150–400/µL), neutrophil percentage 66.9% (normal: 40.0–74.0%), lymphocyte percentage 27.8% (normal: 19.0–48.0%), BUN 12 mg/dL (normal: 7–25/µL), creatinine 0.8 mg/dL (normal: 0.5–0.9 mg/dL), uric acid 4.3 mg/dL (normal: 2.3–7.0 mg/dL), phosphate 3.2 mg/dL (normal: 2.7–4.5 mg/dL), AST 17 U/L (normal: <40 U/L), ALT 11 U/L (normal: <41 U/L), alkaline phosphatase 70 U/L (normal: 35–104 U/L), total bilirubin 0.3 mg/dL (normal: 0.3–1.0 mg/dL), albumin 4.4 g/dL (normal: 3.5–5.7 g/dL), total protein 7.3 g/dL (normal: 6.6–8.7 g/dL), sodium 140 mmol/L (normal: 136–145 mmol/L), potassium 4.0 mmol/L (normal: 3.5–5.1), total calcium 9.1 mg/dL (normal: 8.6–10.2 mg/dL), LDH 170 U/L (normal: 140–271 U/L), TSH 1.33 µIU/mL (normal: 0.25–5.00 µIU/mL), free T4 1.40 ng/dL (normal: 0.89–1.78 ng/dL), T3 126.40 ng/dL (normal: 78.00–182.00 ng/dL). Tests for infections such as human immunodeficiency virus (HIV), hepatitis B virus (HBV), or hepatitis C virus (HCV) all showed negative results. Due to the absence of any prominent complaints like dry eye or dry mouth from the patient, comprehensive rheumatologic examinations, such as testing for anti-SSA/SSB antibodies or sialoscintigraphy, were not conducted.

**Figure 1:**
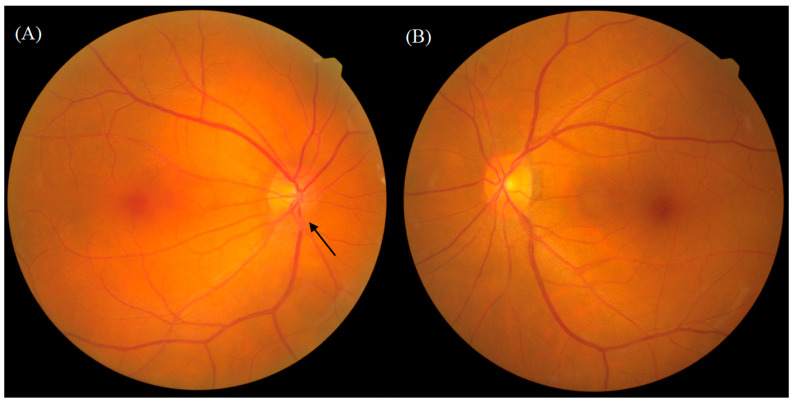
A funduscopic examination demonstrated blurry disc of inferior margin over right eye (black arrow). (**A**) Right eye. (**B**) Left eye.

**Figure 2:**
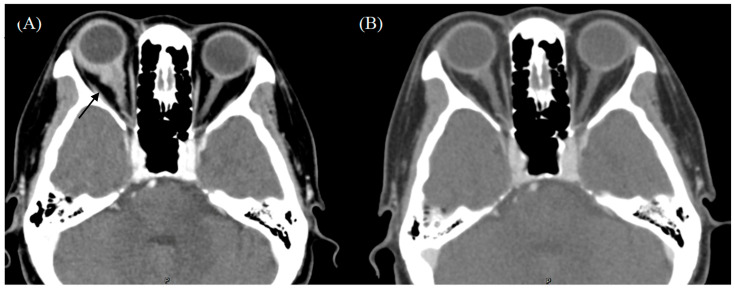
Computed tomography of the orbit demonstrated enhancing soft tissue density in the posterior margin of the right globe and along the right optic nerve. The right globe and optic nerve are compressed, causing exophthalmos (black arrow). (**A**) Before treatment. (**B**) After treatment.

**Figure 3:**
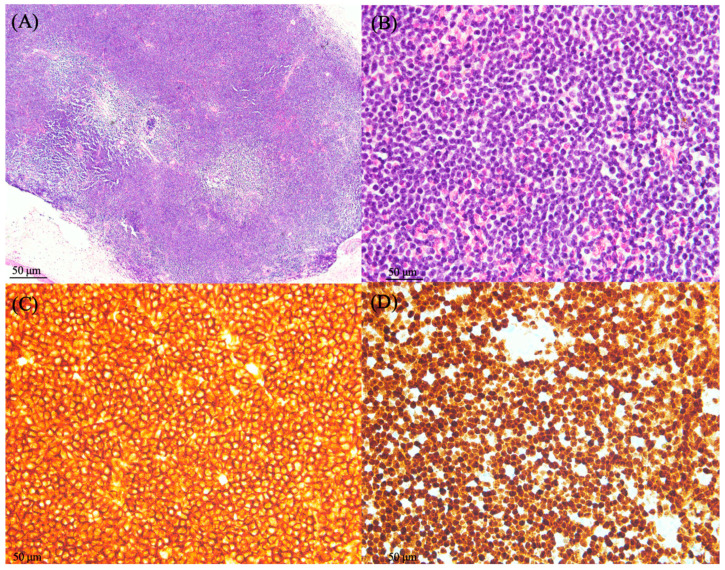
A biopsy of the orbital mass revealed proliferation of small monotonous lymphoid cells without normal architecture of lymphoid tissue in the conjunctival tissue. (**A**) Low power view of H&E revealed submucosal lymphoid aggregation and effacement of follicles (40×). (**B**) High power view showed diffusely small-sized atypical lymphoid cells with monotonous appearance (400×). (**C**,**D**) The positive results of immunohistochemical stains of CD20 and MNDA indicated extranodal marginal zone lymphoma (400×).

The ophthalmologic examination revealed an intraocular pressure of 15 mmHg in the right eye and 14 mmHg in the left eye, with intact bilateral eye movements in all directions. The visual acuity examination revealed an acuity of 6/6 and normal light reflex in the bilateral eyes. A slit-lamp examination revealed right eye chemosis. A funduscopic examination demonstrated a blurry disk of the inferior margin over the right eye (Figure 1). Computed tomography (CT) of the orbit demonstrated enhancing soft tissue density in the posterior margin of the right globe and along the right optic nerve. The right globe and optic nerve are compressed, causing exophthalmos (Figure 2). A biopsy of the orbital mass revealed small monotonous lymphoid cell proliferation without normal lymphoid tissue architecture in the conjunctival tissue. Immunohistochemical staining revealed positive staining for a cluster of differentiation (CD) 20 and myeloid cell nuclear differentiation antigen (MNDA) in the tumor cells (Figure 2). The final diagnosis was localized EMZL of mucosa-associated lymphoid tissue (MALT) after a series of image examinations and biopsy confirmation. There was no Chlamydia psittaci found in the biopsy or cultures from conjunctival swabs and peripheral blood. Under the diagnosis of EMZL in Ann Arbor stage I with the MALT-IPI score presenting as zero, she received six cycles of rituximab, cyclophosphamide, vincristine, and prednisone (R-CVP) therapy every three weeks from May 2022 to September 2022. Complete tumor regression was observed by the follow-up orbital CT scan and she did not complain of ocular-related adverse events (Figure 3). There was not any infection of cytomegavirus (CMV), herpes simplex virus (HSV), or Epstein–Barr virus (EBV) in the serum analysis 3 months after R-CVP therapy. As of February 2024, the patient was in a status of complete remission.

## 3. Discussion

Primary lymphomas of the ocular adnexa region (OAR) represent a rare subset of malignancies characterized by their heterogeneity. They constitute only 1–2% of non-Hodgkin lymphomas (NHLs) and approximately 8% of extranodal lymphomas [1]. EMZL of the ocular adnexa shares similar features with EMZL located at other sites, including general morphology, immunophenotype, and presumptive pathogenesis [5].

The timing of suspicion and consideration for diagnosing ocular lymphoma should be especially heightened in middle-aged or elderly patients who have experienced posterior vitreitis. Similarly, individuals with vitreitis or uveitis that do not respond to steroid treatment or recur after steroid therapy should also raise concerns for ocular lymphoma. Prompt recognition and diagnosis are crucial, particularly in these patient populations, to ensure timely and appropriate management [6]. The initial assessment, which is pivotal in determining the appropriate course of action, encompasses a thorough examination using various diagnostic tools. This includes a slit-lamp examination, ocular ultrasound, fluorescein angiogram, and head MRI. The slit-lamp examination can detect lymphocytic infiltrates in the vitreous or chorioretinal region. Ocular ultrasound may reveal vitreous debris, choroidal thickening, retinal detachment, and an enlarged optic nerve with retrobulbar fat infiltration. A fluorescein angiogram can reveal hyperfluorescent or hypofluorescent punctate lesions, providing crucial insights into the presence of tumor infiltrates within the retinal pigment epithelium. These subtle lesions may not be readily apparent during a routine clinical examination but can be effectively visualized through angiography. If patients present with positive findings during the slit-lamp examination, ocular ultrasound, fluorescein angiogram, or even neurological symptoms suggestive of ocular lymphoma involvement, clinicians may opt to proceed with a brain MRI. This additional diagnostic step aims to comprehensively assess the extent of the disease, particularly in cases where there are indications of central nervous system involvement. By integrating the results of the brain MRI with other diagnostic modalities, clinicians can formulate a more comprehensive understanding of the patient’s condition, facilitating tailored treatment strategies [6,7].

In some cases, patients may present with a medical history encompassing autoimmune disorders, notably thyrotoxicosis (observed in approximately 5% of cases) or Sjögren syndrome. It is imperative to assess the coexistence of these conditions during the diagnostic process, as their presence can potentially exert a detrimental influence on the therapeutic outcomes of extraorbital MALT lymphomas [4]. For patients residing in rural areas who are diagnosed with ocular adnexal mucosa-associated lymphoid tissue (OAML) lymphoma, particular attention should be given to those with a history of chronic conjunctivitis and exposure to domestic animals. It is recommended that these individuals undergo examination for Chlamydia psittaci infection, as there is a recognized association between this pathogen and the development of OAML [5].

In clinical practice, locoregional radiotherapy (RT) is traditionally the first-line treatment of EMZL of mucosa-associated lymphoid tissue at stage I [8]. In general, local RT has demonstrated the ability to achieve high rates of local control, typically falling within the ranges of 90% to 100%, even when systemic disease is concurrently present [9,10]. Additionally, the 5-year disease-relapse-free rate usually exceeds 90% [10,11]. Although RT has been shown to provide excellent local control of OARs in cases of EMZL, the ophthalmologic outcomes may be unfavorable, including visual acuity impairment and deterioration of lens opacity. By the RT dose escalation and accumulation, the patients may suffer from acute adverse effects such as dry eye, conjunctivitis, and momentary periorbital edema [12]. The most common delayed effect was cataract [12]. Symptoms such as glaucoma, retinopathy, and optic nerve injury were also reported [13]. Hence, it is noteworthy to mention that in the year 2013, a retrospective study was conducted, which shed light on the efficacy and tolerability of employing low-dose radiation (specifically 2 × 2 Gy) for the management of orbital lymphoma. The findings of this study revealed that such an approach not only proved to be effective but was also remarkably well tolerated by patients. Moreover, it yielded high response rates, facilitated durable local control of the condition, and was associated with minimal incidence of side effects. However, mild acute side effects, including dry eye, conjunctivitis, and transient periorbital edema, were still observed. In addition, its overall sample size was small and required further study of the low-dose radiation approach with a longer follow-up period [14]. According to the NCCN Guidelines Version 6.2023 of Extranodal MZL of Nongastric Sites [8], a dose of RT of 2 × 2 Gy may be considered in selected patients, such as the elderly or those with Sjögren’s syndrome. Doses of 24 to 36 Gy in 20 fractions are traditional doses and are associated with excellent local control. For patients, multiple sessions of radiation therapy are not a convenient treatment option and may even be burdensome.

Rituximab is a chimeric mouse antihuman CD20 monoclonal antibody. It has been used as immune therapy for lymphoma localized to the ocular adnexa. As of now, the use of rituximab in the treatment of extranodal marginal zone B-cell lymphoma localized to the ocular adnexa has been documented in only a limited number of patients. Within this limited cohort, there appears to be a notable incidence of inadequate periocular response and local recurrence following treatment. The median follow-up times (<31 months) are too short to assess the role of rituximab as therapy for local disease [15,16,17,18]. Among the above treatments of extranodal marginal zone B-cell lymphomas, there were pros and cons in each treatment. When rituximab is used in conjunction with chemotherapy, there is an increase in both response rates and associated toxicities. Since the target lesion of this case is in the ocular region and the side effects of targeted therapy combined with chemotherapy are not difficult to prevent and manage, we preferred treatments with high tumor response rates to avoid possible long-term damage to vision.

According to the literature review, the patients of EMZL of OAR treated by the chemotherapy regimen of cyclophosphamide, vincristine, and prednisolone (CVP) also demonstrated a high response rate and favorable survival outcomes [19]. For the purpose of reducing the toxicity of the standard regimen of the CHOP (cyclophosphamide, doxorubicin, vincristine, and prednisone) protocol, the anthracycline-free R-CVP (rituximab, cyclophosphamide, vincristine, and prednisone) regimen was studied in retrospective and phase II studies, of which gastric EMZL and advanced MZL showed a comparable overall response rate (ORR) and tolerability in both studies [20,21]. The ORR could reach 88%, and the complete response of MZL was 60%. After a median follow-up of 38.2 months, the estimated 3-year progression-free survival (PFS) and overall survival (OS) was 59% and 95%, respectively [20]. Treatment with R-CVP followed by rituximab maintenance has also been investigated in a phase II trial with patients with advanced MZL and EMZL, showing acceptable toxicity profiles with a possible improvement in PFS of 81% at three years compared to that of 59% without maintenance [22].

Though treatment courses at a limited stage of EMZL are 3–4 cycles in most clinical practices [8], a phase III study showed that 6 cycles of R-CHOP/R-CVP presented with an overall response rate of 91% [23]. Based on findings from prior studies, it has been determined that the efficacy of administering R-CHOP therapy every two weeks versus every three weeks does not demonstrate superiority in elderly patients diagnosed with diffuse large B-cell non-Hodgkin lymphoma (DLBCL). Consequently, the standard first-line treatment for previously untreated DLBCL is R-CHOP therapy administered every three weeks [24]. The situation of the treatment interval of EMZL is not discussed by any literature reviews. Therefore, we treated our patient with R-CVP therapy every 3 weeks for six cycles.

The hematologic toxicity of R-CVP included grade 3 or 4 neutropenia (10%) and febrile neutropenia (1.7%). The non-hematologic toxicities were mild and tolerable, such as neuropathy of sensory and motor, alopecia, stomatitis, anorexia, constipation, and asthenia [17]. Nevertheless, this regimen demonstrated favorable tolerability and avoided possible adverse side effects of delayed ophthalmic complications by radiotherapy. Systemic therapy should only be initiated if symptoms are present [25]. Radiotherapy-related visual side effects usually need to be closely monitored, such as conjunctivitis, corneal complications, xerophthalmia, cataracts, visual acuity impairment, or even retinal complications [13]. Given the potential risks associated with radiation therapy, particularly in terms of eye-related complications that could significantly impact quality of life, we opted to manage this case with a regimen of R-CVP chemotherapy instead of pursuing localized radiotherapy. This decision was made after careful consideration of the potential long-term effects and the desire to minimize any adverse impacts on the patient’s overall well-being and quality of life. The primary concern lies in the fact that our patients find it challenging to adhere to radiation therapy sessions scheduled on consecutive days, primarily due to the added inconvenience of daily commuting. This lack of compliance underscores the need for alternative treatment regimens that are more feasible for our patients.

## 4. Conclusions

In conclusion, R-CVP therapy may be one of the effective treatments for ocular EMZL and could avoid possible radiotherapy-related ocular injury in patients of old age and maintain the life quality well.

## Data Availability

Data are contained within the article.
